# Caffeine therapy in preterm infants: effects in 2^nd^ and 3^rd^ childhood

**DOI:** 10.1590/1984-0462/2025/43/2024036

**Published:** 2024-07-08

**Authors:** Johnnatas Mikael Lopes, Nayara Ribeiro Máximo de Almeida, Achilles de Souza Andrade, Victoria Alves Prado

**Affiliations:** a Universidade Federal do Vale São Francisco, Paulo Afonso, BA, Brazil.; b Universidade Federal da Paraíba, João Pessoa, PB, Brazil.

To the Editor,

The immediate postnatal period is the moment for the individual's cardiopulmonary adaptation. It is necessary for the neonate's organic systems to mature so that the hemodynamic and respiratory transformation occurs in a physiological manner.^1^ The cardiopulmonary and neurological immaturity of premature newborns requires caffeine to reduce the need for invasive ventilatory support, facilitate extubation and promote better cognitive outcomes. It is worth noting that the action of caffeine occurs due to its structural similarity to adenosine, acting as a competitive antagonist against the depressant effects of this molecule, through its binding to adenosine A1 and A2A receptors, which leads to beneficial results, especially in cardiac, lung and brain development.^2^

While reading the manuscript "Late effects of caffeine use on sleep of infants born prematurely" by Oliveira et al.,^3^ volume 42, 2024, methodological gaps were found in the analysis of variables regarding wake-up time and total daily sleep time in premature babies undergoing caffeine therapy in the neonatal period.

Therefore, we identified in the study by Oliveira et al.^3^ that the comparison of groups regarding the variables mentioned above is done through variance analysis with Tukey post-hoc, which is considered inadequate due to the imbalance between the groups. This may inflate the type I error due to the post-hoc selection, which should be Hochberg's GT2.^4^

Oliveira et al.^3^ also do not present an effect measure for findings with significant differences, which limits understanding of their clinical usefulness. It is necessary to develop a measure of effect in order to expose the relevance of the findings. Based on this, using the descriptive statistics of the variables "wake-up time" and "total daily sleep time", an effect measure called Cohen's d was created for unbalanced groups.^5^ It reveals that the difference between the preterm (PCG) and at term (TG) groups presents d=1.78, equivalent to a very large effect. The same was evident for the variable "total daily sleep time", with d=1.69.

In view of the above, the considerable differences in effect magnitudes indicate that caffeine administered to neonates may have considerable long-term effects on sleep in childhood. In addition to confirming these findings, it would also be interesting to observe the likely implications related to social interaction and learning that could arise in school-going children due to the longer duration of their sleep. These are the imaginable paths for future investigations into the therapeutic use of caffeine.
