# Supplementary material for: Caffeine therapy in preterm infants: effects in 2nd and 3rd childhood
Source: Rev Paul Pediatr. 2024 Jul 8;43:e2024036. doi: 10.1590/1984-0462/2025/43/2024036 (PMC11251447; doi:10.1590/1984-0462/2025/43/2024036)
Supplement: Related texts

# Reply: Late effects of caffeine use on sleep of infants born prematurely

**Authors:** Ana Carolina Nunes de Oliveira, Ana Paula Cruz de Castro Leão, Ana Lucia Goulart, Allan Chiaratti de Oliveira, Vânia D'Almeida

**Affiliations:** a Universidade Federal de São Paulo, São Paulo, SP, Brasil.

We fully agree with the positive impact of caffeine on the cardiorespiratory function of preterm infants. However, we would like to highlight that its widespread use precedes most of the evidence on the latter positive effects in neurodevelopment.^1,2^ Even though positive neurodevelopment outcomes can be related to caffeine effects beyond adenosine receptor antagonism, this inhibition could have specific and unpredictable effects on sleep neuronal circuitry development, much less explored.^3^

We were surprised by the criticism regarding using the Tuckey *posthoc* test due to different groups’ sample sizes. We use it in normally distributed samples with homogeneous variances between groups. Levene test was applied in both wake-up time (p=0.133) and total daily sleep time (p=0.248) analysis. We recognize the impact of group sample size on the homogeneity of the distributions, but we could not find any references supporting the prioritization of one criterion over the other.

As for the in-between group comparisons, differences observed between individuals born at term (TG) and those born preterm and exposed to caffeine in the neonatal period (PCG) do not equal the caffeine effect.

Although the differences in "wake-up time" and "total daily sleep time" reached statistical significance, we did not interpret these differences as clinically significant, not justifying additional statistical analysis, which we judge, in this case, to be statistical preciosity.

Our results regarding total daily sleep time are in accordance with the normal populational sleep time distribution,^4^ and it is unlikely that in-between group differences in the normal range of sleep time duration would affect social interaction and learning in the studied population.

## References

[1] Kumar P, Walker JK, Hurt KM, Bennett KM, Grosshans N, Fotis MA (2008). Medication use in the neonatal intensive care unit: current patterns and off-label use of parenteral medications. J Pediatr.

[2] Krzyzaniak N, Pawlowska I, Bajorek B (2016). Review of drug utilization patterns in NICUs worldwide. J Clin Pharm Ther.

[3] Gvilia I, Suntsova N, Kostin A, Kalinchuk A, McGinty D, Basheer R (2017). The role of adenosine in the maturation of sleep homeostasis in rats. J Neurophysiol.

[4] Williams JA, Zimmerman FJ, Bell JF (2013). Norms and trends of sleep time among US children and adolescents. JAMA Pediatr.
